# Associations between calcium and magnesium intake and the risk of incident oesophageal cancer: an analysis of the NIH-AARP Diet and Health Study prospective cohort

**DOI:** 10.1038/s41416-020-0818-6

**Published:** 2020-04-03

**Authors:** Shailja C. Shah, Qi Dai, Xiangzhu Zhu, Richard M. Peek, Christianne Roumie, Martha J. Shrubsole

**Affiliations:** 1 Division of Gastroenterology, Hepatology, and Nutrition, Vanderbilt University Medical Center, Nashville, TN USA; 2 Department of Veterans Affairs, Tennessee Valley Healthcare System, Nashville, TN USA; 3 Division of Epidemiology, Vanderbilt Ingram Cancer Center, Vanderbilt University Medical Center, Nashville, TN USA; 4 Department of Medicine, Vanderbilt University Medical Center, Nashville, TN USA

**Keywords:** Risk factors, Epidemiology, Oesophageal cancer, Cancer epidemiology, Cancer prevention

## Abstract

**Background:**

Risk reduction through dietary modifications is an adjunct strategy for prevention of oesophageal cancer, a leading cause of cancer-related mortality and morbidity worldwide. We aimed to estimate the association between calcium and magnesium intakes and incident oesophageal cancer (OC).

**Methods:**

We conducted a retrospective analysis of the NIH-AARP Diet and Health Study prospective cohort. We used multivariable Cox proportional hazard modeling to estimate the association between total intakes and incident OC overall and by histology (oesophageal squamous cell carcinoma (OSCC) and adenocarcinoma (OAC)). Sensitivity and stratified analyses were performed.

**Results:**

Among 536,359 included respondents, 1414 incident OCs occurred over 6.5 million person-years follow-up time. Increasing dietary calcium intake was associated with an adjusted 32–41% lower risk of OSCC compared to the lowest quartile (*p*-trend 0.01). There was a positive association between increasing magnesium intake and OAC risk, but only among participants with low calcium:magnesium intake ratios (*p*-trend 0.04). There was a significant interaction with smoking status.

**Conclusions:**

Based on a retrospective analysis of the NIH-AARP Diet and Health Study prospective cohort, dietary intakes of calcium and magnesium were significantly associated with risk of OSCC and, among certain participants, OAC, respectively. If validated, these findings could inform dietary modifications among at-risk individuals. Mechanistic investigations would provide additional insight.

## Background

Oesophageal cancer (OC) ranks as the 7th most common and 6th most deadly cancer worldwide, responsible for an estimated 572,000 incident cases and 509,000 related deaths annually.^1^ With a <20% 5-year prognosis in the United States (US), OC is responsible for over 16,000 deaths annually (2.6% of all cancer deaths), which is despite an established screening and surveillance program for high-risk groups.^2–4^ The two most common histologic subtypes of OC are oesophageal squamous cell carcinoma (OSCC) and adenocarcinoma (OAC). The geographic variation and differences in predisposing risk factors for these histologic subtypes are notable. Worldwide, OSCC is the predominant histologic subtype and is responsible for over 90% of OC in several lower-income countries, including parts of Asia and Sub-Saharan Africa.^1^ In high-income countries, OAC is the predominant subtype. This variability relates in large part to differences in non-genetic and genetic risk factors. With respect to the former, smoking and alcohol are shared risk factors for both histologic subtypes, although the synergistic effect appears stronger for OSCC. Betel, hot beverages, nitrosamine intake, and likely nutritional deficiencies are also contributory.^5^ Major risk factors for OAC are obesity, gastro-oesophageal reflux, reflux oesophagitis, and Barrett’s oesophagus. Racial, ethnic, and gender differences also exist, as white men are the highest risk demographic for OAC and non-white men (e.g. Asian, blacks) for OSCC.

Research to better define dietary risk factors implicated in the pathophysiology of digestive cancers such as OC and the differential epidemiologic patterns has important implications for decreasing the disease burden, since diet is generally considered a modifiable, low-risk intervention. Calcium (Ca) and magnesium (Mg) are dietary elements that are essential for homeostasis. Altered intake has been associated with a broad range of disease risk, including coronary artery disease, insulin resistance, systemic inflammation, and cancer risk.^6–8^ Ca and Mg are both divalent cations that compete for the same transporters in order to maintain physiologic homeostasis. Few studies analysing the association between Ca or Mg and cancer risk, however, appropriately account for this relationship. Indeed, our group and others have demonstrated that Ca:Mg ratio might be a relevant modifier of the relationship between intake of the single nutrient and disease risk.^9–11^ We recently demonstrated that increasing Ca or Mg dietary intakes were associated with a significantly reduced risk of noncardia gastric adenocarcinoma, even after accounting for these relationships.^12^ With respect to OC specifically, only one study—a case-control study from Ireland—analysed intakes of Ca and Mg and the association with reflux oesophagitis or oesophageal (pre)neoplasia. The authors demonstrated that, especially among individuals with low Ca:Mg intake ratios, the highest Mg intakes were associated with 69–71% lower adjusted odds of reflux oesophagitis and Barrett’s oesophagus, but there was no association with OAC.^8^ OSCC was not evaluated. The association between Ca, Mg and OC has otherwise been incompletely investigated. The vast majority of studies are hospital- or population-based small case-control studies from outside the US that do not account for the relationship between Ca and Mg. These studies are also subject to significant recall bias since the food frequency questionnaires were administered at the time of or after the OC diagnosis. The only prospective study analysing Ca and Mg and OC risk is from Iran and included 47,405 subjects; this study demonstrated that increasing Ca was associated with a significantly reduced risk of OSCC but there was no association with Mg; OAC was not evaluated.^13^ In an early study of the National Institutes of Health-American Association of Retired Persons (NIH-AARP) Diet and Health Study cohort before follow-up was complete, Park et al. analysed the association between dietary and supplemental Ca intakes or dairy intake and the risk of site-specific cancers and demonstrated that increasing dietary Ca intake among men was associated with a decreased risk of OC with a weak non-significant inverse association among women, that was limited by power.^14^ OC histologic subtype was not evaluated separately, nor was the analysis adjusted by Mg intake. There have been no studies analysing the association of Mg and OC among US populations.

Our primary objective was to estimate the association between dietary intakes of Ca and Mg and risk of incident OC stratified by histologic subtype and Ca:Mg ratio in an updated analysis of the NIH-AARP Diet and Health Study prospective cohort.

## Methods

### Study population

The NIH-AARP Diet and Health Study cohort is the largest prospective cohort with dietary data and cancer outcomes in the US. Details of this cohort have been described previously,^15^ but are briefly described here. Between 1995 and 1996, ~3.5 million questionnaires were mailed to AARP members aged 50–71 years who lived in one of eight states or metropolitan areas in the US (6 states: California, Florida, Pennsylvania, New Jersey, North Carolina, Louisiana; and 2 metropolitan areas: Detroit, Michigan and Atlanta, Georgia). Of the baseline questionnaires returned, 566,398 were adequate for entry into the Diet and Health Study cohort based on quality metrics predefined by the NIH-AARP investigators. Date of study entry (T0) was defined as the date of receipt of the baseline questionnaire at the NIH between 1995 and 1996. The cohort follow-up continued prospectively through December 2011, with record linkage for outcome ascertainment detailed below.

### Inclusion and exclusion criteria

**Fig. 1:**
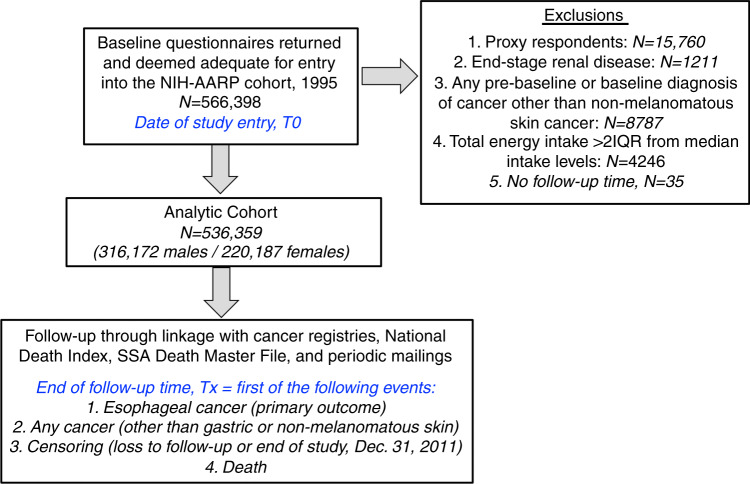
Analytic Cohort Construction. The flow diagram below details the selection criteria which were applied to the original NIH-AARP Diet and Health Study prospective cohort in order to construct the analytic cohort for the present analysis.

Cohort construction with respect to inclusion and exclusion criteria for this study was similar to that previously described.^12^ Briefly, we excluded proxy respondents (*n* = 15 760), those with end-stage renal disease at or prior to T0 (*n* = 1211), those with any diagnosis of cancer other than non-melanomatous skin cancer at or prior to T0 (*n* = 8787) and those with total energy intake (kilocalories (kcal)/day) greater than two interquartile ranges from the sex-adjusted median intake level for the cohort. Thirty-five respondents had negative or zero follow-up time and were excluded. Thus, 536,359 respondents were included for the primary analysis (Fig. 1). Of note, the analytic cohort and their baseline characteristics, including nutritional intakes, are expectedly congruent with our previous study using this cohort,^12^ albeit with some slight differences due to order of application of inclusion/exclusion criteria.

### Exposure assessment: nutritional intakes

The baseline questionnaire was developed at the NCI and included a comprehensive self-administered 124-item semi-quantitative food frequency questionnaire (FFQ), as previously described.^15,16^ This FFQ has been validated for the NIH-AARP cohort using two non-consecutive 24-hour recalls in nearly 2000 participants.^16^ On the FFQ, respondents were asked to estimate their average daily intakes of foods and beverages over the preceding 12 months based on daily frequency and portion size for each item, which was then converted to grams using the DietAARP food database. The food items, standardised frequency and portion categories, and the nutrient database were adapted from the US Department of Agriculture’s (USDA) 1994–1996 Continuing Survey of Food Intake by Individuals (www.ars.usda.gov/ARSUserFiles/80400530/pdf/Dor9496.pdf).

Total dietary Ca and Mg intakes were reported in milligrams according to FFQ responses. Total dietary intake represented the sum of dietary and supplemental intakes. Ca:Mg ratio was calculated as the ratio of total Ca intake (milligrams) to total Mg intake (milligrams). Based on our prior studies in colorectal adenomas and cancer, we used the following previously determined Ca:Mg ratio cut-offs: low Ca:Mg (<1.7), normal Ca:Mg (1.7–2.6), high Ca:Mg (>2.6) ratios.^17,18^

### Study outcome: cancer ascertainment

An incident diagnosis of OC (overall), OAC or OSCC were the primary outcomes for this study. Incident cancer cases were identified through cancer registry linkage, as described previously.^12^ In addition to the original eight cancer registries, starting in 2003 cancer linkage also occurred from three additional states (Arizona, Nevada, and Texas). Linkage to cancer registries is excellent in the NIH-AARP, with ~90% accuracy. As described previously, routine quality checks confirmed that the NIH-AARP Diet and Health Study cancer registry results did not differ significantly from cancer incidence rates based on SEER data for the same states and cancer site classes.^19^ During the study period, each cancer registry was certified by the North American Association of Central Cancer Registries as being at least 90% complete within 2 years of cancer diagnosis. Cancer sites and histologic subtype were identified according to the International Classification of Disease for Oncology, third edition, Surveillance, Epidemiology and End Results (ICD-O-3 SEER) Site/Histology coding system. The ICD-O-3 SEER Site/Histology codes for oesophageal cancers are C15.0–C15.9, which includes all anatomical locations of the oesophagus (C15.0–C15.8) and a “not otherwise specified (NOS)” category (C15.9). Histology codes were used to identify oesophageal squamous cell carcinoma (8041, 8070, 8071, 8072 and 8074), adenocarcinoma (8140, 8142, 8144, 8261, 8310, 8480, 8481 and 8570), and NOS (8010, 8021, 8560).

Follow-up time for the cohort started at the time the baseline questionnaire was received until either OC  diagnosis, any cancer diagnosis other than OC or non-melanomatous skin cancer, loss to follow-up, end of study period (31 December 2011) or death—whichever occurred earliest.^12^ Loss to follow-up was considered if the respondent relocated outside of the cancer registry coverage areas permanently. Death ascertainment was via linkage to the Social Security Administration Death Master file, the National Death Index, and through mailings by the NIH AARP study investigators, as previously described.^12^

### Statistical analysis

Our analytic approach was overall similar to our previous analysis with gastric cancer as the primary outcome.^12^ Ca and Mg intakes were categorised into quantiles. Quartiles were used for most analyses including the primary analysis, while tertiles were used for some stratified analyses to prevent sparse cell counts due to a small number of cases. As described previously,^12^ we presented the baseline variables according to above versus below median Ca and Mg intakes for the cohort. All nutritional intake values were adjusted for age, gender, and energy intake. Student’s *t*-test and Chi-squared tests were used for between-group comparisons of means and frequencies for continuous and categorical variables, respectively. Minimally adjusted (age, gender, race/ethnicity, energy intake) and fully adjusted Cox proportional hazard models were used to estimate hazard ratios (HRs) and 95% confidence intervals (CIs) for risk of incident OC overall, and stratified by histologic subtype.

Factors previously identified or hypothesised to be associated with the exposures (Ca, Mg) or the outcome (OC) were evaluated as potential confounders or effect modifiers. We used categorisation for covariates as previously described.^12^ These covariates of interest included age, gender, race and ethnicity (non-Hispanic white, non-Hispanic black, Hispanic, Asian, Pacific Islander/Alaskan natives/Native Americans/other), obesity (body mass index (BMI, kg/m^2^) ≥ 30), highest level of education achieved (less than high school, completed high school, some post-high school training, or completed college or any postgraduate training), smoking status (never smoker, former smoker, current smoker), self-reported health status, healthy eating index (HEI, continuous score 1–100),^20,21^ and alcohol intake by drinks per day as well as according to gender-specific standards for moderate/heavy alcohol consumption. Covariates which were associated with both the exposure and outcome were included in the multivariable model due to their potential as confounders. These covariates included: race and ethnicity (categorised as non-Hispanic white versus non-white); obesity; smoking status; highest level of education achieved; HEI; and self-reported health status. Similar to our prior study, for models with Ca as the primary exposure, Mg was included as a continuous covariate; for models with Mg as the primary exposure, Ca was included as a continuous covariate.^12^ We adjusted for total daily energy intake using the standard multivariate model method.^22^ Linear dose-response was evaluated by including the quantile as a continuous variable in the model. The lowest quantile was the reference group for all analyses. All covariates had only <5% missing data and thus, records with missing data were excluded from analyses.

Stratified analyses by gender, race/ethnicity, obese status, smoking status, alcohol intake, Ca:Mg ratio, and supplemental versus non-supplemental Ca intake were conducted. Supplemental Mg intake was not separately evaluated because this value was 100 mg for the majority of participants, as reported previously.^12^ To test for interactions, the −2 log(likelihood) test was used to compare models with and without the interaction term of interest. We performed a sensitivity analysis excluding respondents with <12 months of follow-up time, given the possibility that any OCs diagnosed within this time might represent prevalent as opposed to incident cancers. All analyses were conducted in Stata version 15 (StataCorp, Texas, US).

## Results

### Cohort characteristics

A total of 1414 incident cases of OC occurred (996 OAC, 344 OSCC, and 74 histologic subtypes not otherwise specified (NOS)) during 6,503,978 person-years of follow-up time for the cohort. The baseline characteristics, including demographics, lifestyle habits, and nutritional intakes, of the 536,359 respondents comprising the analytic cohort are detailed in Table 1. The majority of participants in the NIH-AARP cohort were non-Hispanic whites with a high school graduate degree or higher. Most participants also reported former or current smoking and moderate-heavy alcohol use by gender-specific standards. More males had below median Ca but above median Mg intakes. Respondents with below median Ca or Mg intakes were more likely to be non-Hispanic whites, ever smokers (low Ca), never smokers (low Mg), moderate/heavy alcohol users (low Ca), and to report lower overall health. Respondents with below median Ca intake had slightly higher average daily age- and gender-adjusted energy intake and those with below median Mg intake had slightly lower average adjusted daily energy intake compared to participants with above median Ca and Mg intakes, respectively. Otherwise, in general, the absolute difference in mean value or distribution for each characteristic was similar irrespective of Ca or Mg intake. Because of the very large cohort size, clinically similar values reached statistical significance.

**Table 1:** Cohort characteristics stratified by median calcium and magnesium total intakes, NIH-AARP Diet and Health Study 1995–2011.

Characteristic	Calcium Intake (Median:881.3 mg)			Magnesium Intake (Median: 358.6 mg)		
	Below Median (*N* = 268,180)	Above Median (*N* = 268,179)	*P*-value	Below Median (*N* = 268,162)	Above Median (*N* = 268,197)	*P*-value
Age at entry (mean, SD), years	61.6 (5.4)	61.7 (5.4)	<0.001	61.7 (5.4)	61.6 (5.4)	<0.001
Male (%)	63.9%	54.0%	<0.001	52.0%	65.9%	<0.001
Race/Ethnicity (%)			<0.001			<0.001
Non-Hispanic white	91.7%	93.6%		92.0%	93.2%	
Non-Hispanic black	4.6%	3.1%		4.4%	3.4%	
Hispanic	1.9%	1.9%		1.9%	1.9%	
Asian	1.4%	1.0%		1.4%	1.1%	
Pacific Is/Alaskan/ Native Am/Other	0.4%	0.4%		0.4%	0.4%	
BMI (mean, SD), m/kg^2^	27.2 (5.0)	27.0 (5.2)	<0.001	27.1 (5.2)	27.1 (5.1)	0.70
Obese status (BMI > 30), (%)	24.3%	23.6%	<0.001	24.3%	23.6%	<0.001
Smoking status (%)			<0.001			<0.001
Never smoker	34.5%	38.0%		38.0%	34.5%	
Former smoker	52.2%	50.5%		49.4%	53.3%	
Current smoker	13.3%	11.5%		12.6%	12.2%	
Alcohol use (%)			<0.001			<0.001
None	23.8%	25.3%		26.0%	23.1%	
Up to 1 drink/day (but not 0 g/day)	18.7%	19.9%		21.9%	16.7%	
Up to 2 drinks/day	8.8%	8.5%		9.2%	8.1%	
2–3 drinks/day	6.4%	6.3%		6.6%	6.1%	
>3 drinks/day	42.2%	40.1%		36.2%	46.1%	
Moderate/heavy alcohol use, gender-specific standards (%)	51.6%	50.2%	<0.001	46.8%	54.9%	<0.001
Highest education achieved (%)			<0.001			<0.001
Less than high school	27.3%	25.3%		28.1%	24.5%	
Completed high school	10.2%	10.1%		10.2%	10.0%	
Some post-high school training	23.8%	24.0%		24.3%	23.5%	
Completed college and/or postgraduate	38.7%	40.7%		37.4%	42.0%	
Self-reported health (%)			<0.001			<0.001
Excellent or very good	51.0%	53.1%		50.6%	53.4%	
Good	35.8%	34.1%		35.9%	34.0%	
Fair or Poor	13.2%	12.8%		13.5%	12.6%	
Daily energy intake^a^ (mean, SD), kcal/day	1869.2 (222.4)	1824.1 (230.9)	<0.001	1815.0 (230.3)	1878.4 (220.8)	<0.001
Healthy Eating Index Score^b^ (mean, SD)	68.1 (1.6)	67.2 (2.1)	<0.001	68.5 (1.4)	66.7 (2.0)	<0.001
Total calcium intake^b^ (mean, SD), mg	899.1 (218.5)	1139.7 (305.8)	<0.001	877.1 (193.5)	1161.8 (303.8)	<0.001
Dietary, total (mean, SD), mg	668.3 (205.7)	887.3 (320.1)		618.2 (165.6)	937.5 (300.5)	
Dairy source (mean, SD), mg	369.6 (116.0)	494 (180.0)		342.0 (93.7)	521.6 (169.5)	
Nondairy source (mean, SD), mg	298.8 (89.9)	393.2 (140.2)		276.2 (72.2)	415.9 (131.2)	
Supplemental intake (mean, SD), mg	545.9 (37.2)	699.8 (74.1)		570.1 (55.3)	536.4 (58.6)	
Calcium supplement (yes), %	30.2%	71.9%	<0.001	54.8%	55.6%	<0.001
Total magnesium intake^b^ (mean, SD), mg	335.8 (82.3)	421.3 (128.3)	<0.001	314.7 (66.1)	442.5 (119.8)	<0.001
Dietary, total (mean, SD), mg	291.7 (84.4)					
Supplemental intake (mean, SD), mg	93.2 (1.5)	93.7 (1.9)		93.0 (1.4)	93.8 (2.0)	
Magnesium supplement (yes), %	40.3%	65.0%	<0.001	36.4%	69.0%	<0.001

^a^Adjusted for age, gender.

^b^Adjusted for age, gender, race/ethnicity, energy intake.

A minority of men and women achieved the age- and gender-specific Recommended Dietary Allowance (RDA) for Ca (11.2% women, 24.7% men) and Mg (34.7% women, 25.2% men) in the cohort. Nearly three-quarters of women (73.7%) and <40% of men (38.9%) overall reported ever taking Ca-containing supplements; daily use was reported in 67% of women and 53% of men. Over half of all participants reported taking Mg-containing supplements with 71% of these individuals reporting taking 100 mg of supplemental Mg daily.

### Association of Ca and Mg intakes with oesophageal cancer

Increasing Ca was inversely associated with OC in the minimally (*p*-trend = 0.04) but not fully adjusted models; the associations between Ca intake and risk of OAC were also null (Table 2). The associations between Mg intake and OC, including by histologic subtype were overall null, but there was evidence of significant effect modification according to Ca:Mg ratio and smoking status, as detailed below.

**Table 2:** Associations between calcium and magnesium total intakes and risk of oesophageal cancer (OAC, OSCC, total), NIH-AARP Diet and Health Study 1995–2011.

Total follow-up time: 6,503,978 person-years	Quartiles of Calcium or Magnesium intake								*p*-trend
	Quartile 1 (Low) Ca, mean (SD): 440 (110) mg Mg, mean (SD): 215 (45) mg		Quartile 2 Ca, mean (SD): 737 (80) mg Mg, mean (SD): 318 (24) mg		Quartile 3 Ca, mean (SD): 1068 (119) mg Mg, mean (SD): 404 (29) mg		Quartile 4 (High) Ca, mean (SD): 1833 (515) mg Mg, mean (SD): 578 (124) mg		
Cancer type	Cases	HR, 95% CI	Cases	HR, 95% CI	Cases	HR, 95% CI	Cases	HR, 95% CI	
**Oesophageal, Total (1414 cases)**^a^									
**Calcium**	**369**		**395**		**349**		**301**		
Minimally adjusted^b^		1.00 (ref)		0.96 (0.82–1.11)		0.85 (0.72–1.01)		0.84 (0.68–1.02)	**0.04**
Fully adjusted ^b^		1.00 (ref)		1.00 (0.86–1.17)		0.93 (0.78–1.11)		0.98 (0.79–1.21)	0.65
**Magnesium**	**301**		**346**		**365**		**402**		
Minimally adjusted^b^		1.00 (ref)		0.98 (0.83–1.15)		0.91 (0.77–1.08)		0.83 (0.67–1.02)	0.07
Fully adjusted^b,c^		1.00 (ref)		1.06 (0.89–1.26)		1.07 (0.89–1.28)		1.09 (0.86–1.37)	0.50
**OAC (996 cases)**									
**Calcium**	**246**		**293**		**253**		**204**		
Minimally adjusted^b^		1.00 (ref)		1.12 (0.94–1.34)		1.05 (0.86–1.29)		1.06 (0.83–1.35)	0.81
Fully adjusted^b,c^		1.00 (ref)		1.16 (0.96–1.40)		1.10 (0.89–1.37)		1.17 (0.91–1.51)	0.34
**Magnesium**	**204**		**252**		**260**		**280**		
Minimally adjusted^b^		1.00 (ref)		1.05 (0.87–1.26)		0.96 (0.78–1.18)		0.91 (0.70–1.17)	0.35
Fully adjusted^b,c^		1.00 (ref)		1.18 (0.96–1.45)		1.18 (0.95–1.47)		1.28 (0.96–1.69)	0.11
**OSCC (344 cases)**									
**Calcium**	**108**		**83**		**76**		**77**		
Minimally adjusted^b^		1.00 (ref)		0.63 (0.47–0.85)		0.49 (0.35–0.68)		0.44 (0.29–0.65)	**<0.001**
Fully adjusted^b,c^		1.00 (ref)		0.68 (0.48–0.92)		0.59 (0.42–0.84)		0.61 (0.41–0.92)	**0.01**
**Magnesium**	**81**		**80**		**86**		**97**		
Minimally adjusted^b^		1.00 (ref)		0.90 (0.65–1.24)		0.90 (0.64–1.26)		0.72 (0.47–1.12)	0.20
Fully adjusted^b,c^		1.00 (ref)		0.92 (0.66–1.30)		0.94 (0.65–1.36)		0.87 (0.54–1.39)	0.62

^a^“Total oesophageal cancer” includes OAC (*n* = 996), OSCC (*n* = 344) and oesophageal cancer histologic subtype not otherwise specified (NOS) (*N* = 74).

^b^Adjusted for age, gender, race/ethnicity, energy intake.

^c^Additionally adjusted for obesity, smoking, alcohol intake, self-reported health, healthy eating index (HEI), educational status and total Ca (or Mg) intake.

Bold values indicate statistical significance *p* < 0.05.

There was a strong inverse association between increasing Ca intakes and risk of OSCC on both minimally and fully adjusted models (*p*-trend < 0.001 and 0.01, respectively). Compared to the lowest quartile of Ca intake, higher intakes of Ca were inversely associated with risk of OSCC (*p*-trend = 0.01) and strongest for quartile 3 (HR 0.59, 95% CI: 0.42–0.84). Excluding respondents with less than 12 months follow-up time (*n* = 5508, of whom 31 were diagnosed with OC—15 OAC, 15 OSCC, 1 OC NOS) did not significantly affect the findings (Supplementary Table 1S, sensitivity analysis).

### Stratified analyses

Smoking status demonstrated a stronger positive association between Mg and OAC among never-smokers compared to ever-smokers, with the highest quartile of Mg intake associated with a 2.5–fold higher adjusted risk of OAC compared to the lowest quartile (HR 2.51, 95% CI: 1.26–4.97; *p*-trend 0.003), with a statistically significant interaction (p-interaction = 0.03). When stratified by smoking status, the inverse association between Ca and OSCC was preserved among ever-smokers (*p* = 0.003) and null for never-smokers (*p* = 0.79); however, there was no evidence of statistically significant interaction (*p*-interaction = 0.32). (Supplementary Table 2S)

Among participants with low Ca:Mg intake ratios (<1.7), Mg intake in the highest versus lowest tertile was associated with a 1.96-fold significantly higher adjusted risk of OAC (HR 1.96, 95% CI: 1.01–3.77). The inverse association between Ca intake and OSCC was maintained for low Ca:Mg ratios (HR 0.33, 95% CI: 0.07–1.67 for highest vs lowest tertile of Ca intake), but not for high-normal ratios; however, these associations and linear trends were not statistically significant and likely reflect reduced power within strata. There were otherwise no substantive differences on stratified analysis by Ca:Mg ratio (Table 3).

**Table 3:** Associations between calcium and magnesium total intakes and risk of oesophageal (OAC, OSCC, total) stratified by Ca:Mg ratio^a^, NIH-AARP Diet and Health Study 1995–2011.

	Tertiles of Calcium or Magnesium Intake							
	Tertile 1 (Low) Ca, mean (SD): 491 (132) mg Mg, mean (SD): 234 (51) mg		Tertile 2 Ca, mean (SD): 890 (125) mg Mg, mean (SD): 360 (33) mg		Tertile 3 (High) Ca, mean (SD): 1677 (521) mg Mg, mean (SD): 543 (124) mg		*p*-trend	*p*-interaction
	Cases	HR, 95% CI	Cases	HR, 95% CI	Cases	HR, 95% CI		
**OAC (996 cases)**								
**Calcium**								
Overall^b^		1.00 (ref)		1.07 (0.90–1.27)		1.20 (0.97–1.48)	0.10	
Ca:Mg < 1.7 (171)	**127**	1.00 (ref)	**42**	1.03 (0.61–1.75)	**2**	0.17 (0.02–1.50)	0.65	(ref)
Ca:Mg ≥ 1.7, <2.6 (480)	**194**	1.00 (ref)	**216**	1.02 (0.77–1.34)	**70**	1.08 (0.64–1.81)	0.82	0.99
Ca:Mg ≥ 2.6 (345)	**23**	1.00 (ref)	**98**	0.99 (0.60–1.61)	**224**	1.13 (0.67–1.91)	0.44	0.68
**Magnesium**								
Overall^b^		1.00 (ref)		1.13 (0.95–1.36)		1.18 (0.94–1.49)	0.16	
Ca:Mg < 1.7 (171)	**52**	1.00 (ref)	**58**	1.43 (0.90–2.26)	**61**	1.96 (1.01–3.77)	**0.04**	(ref)
Ca:Mg ≥ 1.7, <2.6 (480)	**143**	1.00 (ref)	**168**	1.21 (0.92–1.59)	**169**	1.40 (0.93–2.10)	0.10	0.50
Ca:M ≥ 2.6 (345)	**91**	1.00 (ref)	**117**	1.06 (0.77–1.43)	**137**	0.99 (0.66–1.48)	0.95	0.97
**OSCC (344 cases)**								
**Calcium**								
Overall^b^		1.00 (ref)		0.81 (0.60–1.08)		0.77 (0.54–1.10)	0.15	
Ca:Mg < 1.7 (106)	**77**	1.00 (ref)	**25**	0.69 (0.33–1.43)	**4**	0.33 (0.07–1.67)	0.19	(ref)
Ca:Mg ≥ 1.7, <2.6 (126)	**45**	1.00 (ref)	**58**	1.29 (0.75–2.21)	**23**	1.54 (0.59–4.07)	0.34	0.18
Ca:Mg ≥ 2.6 (112)	**7**	1.00 (ref)	**30**	0.99 (0.42–2.34)	**75**	1.34 (0.55–3.29)	0.30	0.19
**Magnesium**								
Overall^b^		1.00 (ref)		1.04 (0.61–1.77)		1.16 (0.58–2.35)	0.69	
Ca:Mg < 1.7 (106)	**37**	1.00 (ref)	**34**	1.06 (0.60–1.86)	**35**	0.73 (0.31–1.73)	0.57	(ref)
Ca:Mg ≥ 1.7, <2.6 (126)	**34**	1.00 (ref)	**44**	1.15 (0.68–1.94)	**48**	1.08 (0.51–2.30)	0.81	0.48
Ca:Mg ≥ 2.6 (112)	**33**	1.00 (ref)	**37**	1.04 (0.61–1.77)	**42**	1.16 (0.58–2.35)	0.61	0.97

^a^Tertile was selected for exposure categorization to prevent sparse cell counts due to a small number of cases.

^b^All models adjusted for age, gender, race/ethnicity, energy intake, obesity, smoking, alcohol intake, self-reported health, healthy eating index (HEI), educational status, and total Ca (or Mg) intake.

Bold values indicate statistical significance *p* < 0.05.

There was no evidence of significant effect modification by gender, race/ethnicity, obesity status, or supplemental vs non-supplemental source of intake on stratified analyses, although low counts did limit robust analysis of race/ethnicity. There was no statistically significant interaction of these covariates on the associations between Ca and Mg intakes and OC, OAC, OSCC (all *p*-interactions > 0.05).

## Discussion

Our analysis of 536,359 participants of the NIH-AARP Diet and Health Study prospective cohort represents the largest and most comprehensive analysis of the associations between Ca and Mg intakes and the risk of incident OC and the histologic subtypes, OSCC and OAC. Stratification by histologic subtype as well as by Ca:Mg ratio in our study unmasked differences in the associations between Ca and Mg intakes and the risk of OSCC and OAC. We demonstrated that, after adjusting for relevant confounders, increasing levels of dietary Ca intake were associated with a 32–41% significantly lower risk of OSCC compared to the lowest quartile of Ca intake. There was a nearly 2-fold higher adjusted risk of OAC among participants with Mg intakes in the highest versus lowest tertile of Mg intake, but only for those with low Ca:Mg intake ratios (*p*-trend = 0.04). Smoking status modified these associations. Otherwise, associations between Ca, Mg and incidence of OC in the NIH-AARP cohort were generally null.

The only other prospective cohort analysis analysing both Ca and Mg and the risk of OC, which was conducted in Iran among 47,405 subjects, similarly demonstrated a significant inverse association between increasing Ca intakes and risk of OSCC (adjusted HR 0.49, 95% CI: 0.29–0.82 for the highest versus lowest quartile of Ca intake, *p*-trend = 0.01), and found no association between Mg intake and OSCC.^13^ OAC was not evaluated in this study, likely due to insufficient case numbers, since OSCC is the predominant histologic subtype in the region. Among other differences the authors did not adjust for Mg or evaluate for effect modification by Ca:Mg intake. An earlier study of the NIH-AARP cohort with follow-up only through 2003 (547 OC cases total, including 468 males, 79 females) reported an inverse association between dietary Ca intake and OC overall among men but not women, the latter of which could reflect insufficient power; however, the authors did not report on OSCC and OAC separately nor did they consider Mg or the Ca:Mg intake ratio.^14^ We similarly found an inverse association between increasing Ca intake and OC overall, but not after adjusting for confounders. Otherwise, there are only a handful of small hospital- or population-based case-control studies analysing dietary Ca (Mg not analysed) and the odds of OC overall or by histologic subtype (OSCC vs OAC). The results of these studies are mixed, which is likely related to different geographic regions/populations, inclusion criteria, and analytic approach, including variable effort adjusting for confounders, among others. A recent meta-analysis of these studies did demonstrate an overall inverse association between dietary Ca and likelihood of OC, which was driven by the inverse association with OSCC, as there was no association between dietary Ca intake and OAC; importantly this inverse association was only observed in studies from Asia, but not those conducted in European or American populations.^23^ A Swedish cohort study demonstrated a suggestive positive association between albumin adjusted-serum Ca and incident OC (p-trend 0.05), but did not report results stratified by OSCC vs OAC.^24^ Importantly, serum Ca is tightly regulated physiologically and does not reflect dietary intake or total body stores. Instead, the major determinant of total body Ca balance is dietary intake, as evaluated in the present study.^24,25^

Few studies have evaluated associations between Mg intake and OC risk. A population-based case-control study from northern Ireland found that study subjects with the highest versus lowest dietary Mg intake had significantly *reduced* odds of reflux esophagitis and Barrett’s oesophagus, a precursor lesion for OAC, which was most pronounced among those with low Ca:Mg intake ratios; however, there was no association between Ca or Mg and odds of OAC (OSCC not analysed).^8^ Our findings among the NIH-AARP cohort are in contrast with that study, as we report here a nearly 2-fold higher adjusted odds of OAC among individuals with Mg intakes in the highest vs lowest tertile, but only in those with low Ca:Mg intake ratios. The reasons for this discrepancy are not clear but might relate to US vs Irish population differences or study design. Two critical limitations of case-control studies, which are overcome with our prospective cohort study design, must be emphasised: recall bias and reverse causality—that is, the OC diagnosis modifying nutritional intakes of Ca and Mg. Further, the heterogeneity of these studies with respect to population, design, selection of controls, exposure and outcome measurements, and statistical approach, particularly controlling for confounders, possibly underlies the mixed results.

While the Western diet is energy replete with obesity reaching epidemic proportions, key nutrient deficiencies are increasingly recognised and might predispose to both benign and malignant diseases. Our finding that only a minority of participants met the RDA for Ca or Mg intakes is congruent with findings from the National Health and Nutrition Examination Survey (NHANES)^26^ data and the USDA.^27^ In fact, based on NHANES data, <10% of women 51 years or older met adequate dietary Ca intakes and only a minority of men and women achieved adequate recommended intakes of Ca, even when supplemental Ca was considered. As previously noted, our group and others have established the importance of Ca:Mg intake ratio as a potential effect modifier of the association between Ca or Mg and the disease of interest, including other gastrointestinal tract neoplasia.^12,17,18,28^ Dietary Ca:Mg ratio modulates systemic inflammation and has been implicated in insulin resistance, cardiovascular disease, and, relevant to our study, downstream pathways that promote oncogenesis if aberrant.^29–31^ Ca:Mg ratio patterns also demonstrate geographic variability even if intake of Ca or Mg demonstrates geographic similarity. For example, US populations overall generally have adequate to high Ca:Mg ratios, while East Asian populations generally have adequate to low Ca:Mg ratios,^9^ which might suggest underlying genetic or gene-environment influences. Defining mechanisms of how the Ca:Mg intake ratio modulates carcinogenesis, as well as the mechanisms underlying other modifying factors such as smoking and geography, might have relevant translational impact with respect to cancer prevention and risk attenuation.

This study has several strengths. Well-designed nutritional intake studies defining associations between dietary intakes and cancer risk, especially digestive tract cancers, are worthwhile adjuncts for cancer prevention since diet is modifiable. Unfortunately, there are important logistical considerations for nutritional intake studies in general, including accuracy and reliability of exposure and outcome measurement, duration and completeness of follow-up, response rate to validated questionnaires, cost, and appropriate power for rarer outcomes such as cancer. Our use of the  NIH-AARP Diet and Health Study prospective cohort provided a large number of OC cases stratified by histologic subtype and ensured our analyses were appropriately powered. Prior studies either only analysed one histologic subtype, analysed OC overall, or included small numbers of OAC and OSCC cases. Further adding to this study’s rigor are minimal missing data, excellent linkage to cancer registry data and death determination with confirmed accuracy. The prospective design eliminates the potential for recall bias or reverse causality. Because OC was the first primary cancer for all cases in this study, detection bias—which might result from increased endoscopic surveillance from prior head and neck or gastric cancer diagnosis, for example—was also not a concern. That the sensitivity analysis removing participants with <12 months of follow-up did not alter our findings further mitigates concerns for potential reverse causality, i.e. altered dietary intake due to clinical manifestations of a prevalent, and not incident, OC. Nevertheless, some limitations must be acknowledged, particularly generalisability. The NIH-AARP Diet and Health Study cohort is largely comprised of well-educated non-Hispanic whites. Notably, a very large percentage of the cohort reported moderate to heavy alcohol use based on gender-specific standards, which is relevant for this study given the increased risk of OC associated with alcohol use. Only a minority achieved the RDA for Ca or Mg intakes, which is consistent with other population-based studies in the US.^27,32^ Geographic and cultural differences, including dietary preferences, might similarly limit generalisability, and this might further be impacted by underlying host genetics and gene-environment interactions. Small case numbers for some strata, including by race/ethnicity, limited rigorous evaluation for differences. Lastly, an inherent limitation of FFQ-based studies is that it is not possible to confirm whether participants’ responses on a single FFQ adequately reflect future intakes over the study period. This may have resulted in non-differential misclassification, which could bias results towards the null.

In summary, dietary intakes of Ca and Mg are significantly associated with risk of OSCC and, among certain participants, OAC, respectively. External validation studies in other populations, both US-based and non-US-based are needed. Experimental studies investigating aetiologies for the findings reported here will serve as important adjuncts to extend our understanding of OC biology and to define preventive and risk attenuating interventions. Indeed, OC remains one of the most prevalent and deadly cancers globally and highlights the immediate need for achievable efforts.

## Supplementary information

Supplemental Material

## Data Availability

The data that support the findings of this study are available from the NIH-AARP Diet and Health Study. Restrictions apply to the availability of these data, which were used under license for this study. Data are available with the permission of the NIH AARP.
