# Supplementary material for: Associations between calcium and magnesium intake and the risk of incident oesophageal cancer: an analysis of the NIH-AARP Diet and Health Study prospective cohort
Source: Br J Cancer. 2020 Apr 3;122(12):1857–64. doi: 10.1038/s41416-020-0818-6 (PMC7283350; doi:10.1038/s41416-020-0818-6)
Supplement: Supplementary file 1 — Supplemental Material [file 41416_2020_818_MOESM1_ESM.docx]

**SUPPLEMENTAL TABLES:**

**Table 1S (Sensitivity Analysis): Associations between calcium and magnesium total intakes and oesophageal cancer (OAC, OSCC, total) among those with at least 12 months follow-up**^a^**, NIH-AARP Diet and Health Study 1995-2011**

|  | **Quartiles of Calcium or Magnesium Intake** | | | | | | | | **p-trend** |
| --- | --- | --- | --- | --- | --- | --- | --- | --- | --- |
|  | **Quartile 1 (Low)**  Ca, mean (SD): 440 (111) mg  Mg, mean (SD): 215 (45) mg | | **Quartile 2**  Ca, mean (SD): 734 (80) mg  Mg, mean (SD): 318 (24) mg | | **Quartile 3**  Ca, mean (SD):1068 (119) mg  Mg, mean (SD): 404 (28) mg | | **Quartile 4 (High)**  Ca, mean (SD): 1833 (514) mg  Mg, mean (SD): 578 (124) mg | |  |
|  | **Cases** | **HR, 95% CI** | **Cases** | **HR, 95% CI** | **Cases** | **HR, 95% CI** | **Cases** | **HR, 95% CI** |  |
| **Oesoph. Ca, total (1383)***  ***CALCIUM*** | **360** | 1.00 (ref) | **391** | 1.02 (0.87-1.19) | **339** | 0.92 (0.77-1.11) | **293** | 0.98 (0.79-1.21) | 0.62 |
| ***MAGNESIUM*** | **295** | 1.00 (ref) | **337** | 1.06 (0.89-1.26) | **359** | 1.08 (0.90-1.30) | **392** | 1.11 (0.88-1.41) | 0.37 |
|  |  |  |  |  |  |  |  |  |  |
| **OAC (981)**  ***CALCIUM*** | **243** | 1.00 (ref) | **291** | 1.16 (0.96-1.41) | **247** | 1.08 (0.87-1.34) | **200** | 1.16 (0.89-1.49) | 0.42 |
| ***MAGNESIUM*** | **202** | 1.00 (ref) | **247** | 1.17 (0.95-1.43) | **257** | 1.18 (0.95-1.48) | **275** | 1.28 (0.97-1.70) | 0.11 |
|  |  |  |  |  |  |  |  |  |  |
| **OSCC (329)**  ***CALCIUM*** | **102** | 1.00 (ref) | **81** | 0.69 (0.50—0.95) | **72** | 0.59 (0.41-0.85) | **74** | 0.63 (0.41-0.95) | **0.02** |
| ***MAGNESIUM*** | **77** | 1.00 (ref) | **76** | 0.93 (0.65-1.32) | **83** | 0.97 (0.67-1.41) | **93** | 0.93 (0.58-1.51) | 0.85 |
|  |  |  |  |  |  |  |  |  |  |

^a^Models adjusted for age, gender, race, energy intake, smoking, obesity, alcohol intake, self-reported health, HEI, educational status, and calcium (or magnesium) intake.

**Note:* There were 5,508 participants with <12mo follow up; of these, 15 participants had OAC, 15 OSCC, and 1 had oesophageal cancer NOS.

**Table 2S: Associations between calcium and magnesium total intakes and risk of oesophageal cancer (OAC, OSCC) stratified by smoking status^a^, NIH-AARP Diet and Health Study 1995-2011**

|  | **Quartiles of Calcium or Magnesium Intake** | | | | | | | |  |  |
| --- | --- | --- | --- | --- | --- | --- | --- | --- | --- | --- |
|  | **Quartile 1 (Low)**  Ca, mean(SD): 440(111)mg  Mg, mean (SD): 215(45) mg | | **Quartile 2**  Ca, mean (SD): 737(80) mg  Mg, mean (SD): 318(24) mg | | **Quartile 3**  Ca, mean (SD): 1068(119) mg  Mg, mean (SD): 404(29) mg | | **Quartile 4 (High)**  Ca, mean (SD): 1833(515) mg  Mg, mean (SD): 578(124) mg | |  |  |
|  | **Cases** | **HR, 95% CI** | **Cases** | **HR, 95% CI** | **Cases** | **HR, 95% CI** | **Cases** | **HR, 95% CI** | **p-trend** | **p-interaction** |
| **OAC (952 Cases)**  ***CALCIUM*** |  |  |  |  |  |  |  |  |  |  |
| **Ever-smoker** (781) | **202** | 1.00 (ref) | **236** | 1.16 (0.94-1.42) | **192** | 1.05 (0.83-1.33) | **151** | 1.11 (0.83-1.47) | 0.70 | 0.19 |
| **Never-smoker** (171) | **34** | 1.00 (ref) | **45** | 1.19 (0.73-1.93) | **48** | 1.41 (0.85-2.35) | **44** | 1.47 (0.81-2.67) | 0.17 |  |
| ***MAGNESIUM*** |  |  |  |  |  |  |  |  |  |  |
| **Ever-smoker** (781) | **165** | 1.00 (ref) | **203** | 1.13 (0.90-1.41) | **193** | 1.04 (0.81-1.32) | **220** | 1.13 (0.83-1.53) | 0.66 | **0.03** |
| **Never-smoker** (171) | **27** | 1.00 (ref) | **39** | 1.48 (0.87-2.53) | **53** | 2.26 (1.31-3.91) | **52** | 2.51 (1.26-4.97) | **0.003** |  |
|  |  |  |  |  |  |  |  |  |  |  |
| **OSCC (322 Cases)**  ***CALCIUM*** |  |  |  |  |  |  |  |  |  |  |
| **Ever-smoker** (279) | **92** | 1.00 (ref) | **65** | 0.60 (0.43-0.84) | **56** | 0.48 (0.32-0.70) | **66** | 0.54 (0.35-0.84) | **0.003** | 0.32 |
| **Never-smoker** (43) | **8** | 1.00 (ref) | **10** | 1.29 (0.48-3.50) | **15** | 1.77 (0.65-4.81) | **10** | 1.07 (0.32-3.56) | 0.79 |  |
| ***MAGNESIUM*** |  |  |  |  |  |  |  |  |  |  |
| **Ever-smoker** (279) | **65** | 1.00 (ref) | **61** | 0.88 (0.60-1.27) | **68** | 1.01 (0.68-1.49) | **85** | 0.99 (0.60-1.64) | 0.88 | 0.65 |
| **Never-smoker** (43) | **5** | 1.00 (ref) | **5** | 1.16 (0.50-2.73) | **9** | 0.64 (0.23-1.78) | **3** | 0.40 (0.10-1.63) | 0.15 |  |
|  |  |  |  |  |  |  |  |  |  |  |

^a^Models adjusted for age, gender, race, energy intake, obesity, alcohol intake, self-reported health, HEI educational status, and calcium (or magnesium) intake.

^*Note*: Smoking status was missing for 44 OAC and 22 OSCC; these cases were excluded from the stratified analysis
